# Endorsed Hospitals

**Published:** 1916-12

**Authors:** 


### Endorsed Hospitals.

The Annual Report of the Chief of the Medical and Surgical Staff of St. Luke's Hospital, in Utica, New York, to its Board of Trustees, makes an argument regarding the relation of the small endowed, or semi-endowed private hospital, to the community that supports them. That there is such a responsibility, no one can deny. He quotes from Dr. Winford Smith, superintendent of Johns Hopkins Hospital, as follows:

"In this country it can be said without fear of contradiction, that the best hospitals and those that have contributed most up to the present time, to medical education and scientific work, and which have been managed most efficiently in the interests of the patients, have been endowed hospitals." (From the Journal of the American Medical Association, Oct. 1915).

"It is too true that in those hospitals supported by municipalities or by the State, politics both in the selection of men who shall do the work, as well as in the equipment which the best men must have in these days to make a hospital successful, interferes so largely with the management that the best men prefer to work in private or semi-private hospitals. The day has come, however, where a certain amount of research work ought to be done in all hospitals, where the statistics are sufficiently large to make even a small contribution to the science of medicine—because this is, after all, the contribution which a hospital should make to the community. If a hospital is supported by private subscription of a large number of people, there should be some return to the community that these subscriptions represent which is, in a way, adequate. Such must be the case, or else ultimately the private hospital will fail of its support.

"Research work, however, cannot be continued on any considerable scale without the expenditure of more money than most hospitals, even those of endowments, can afford. In large cities it comes about that the hospitals may be so grouped in their interests that the research work may be done in especially constructed institutions, the material for the work being furnished by the hospital, and the support of these institutions being either municipal or in a few instances by large endowments.

"At the present time the hospital that contributes most to the community and is of the most importance to the profession is the one in which the most accurate diagnosis are made. It is to this great middle class of people that the well organized hospital may contribute most. It is not necessary that formal consultations of a large number of well trained men who look over the cases and give opinions regarding them should be frequent, but a staff of men who have the bond of friendship and the sense of esprit de corps, and the opportunity to frequently meet and discuss matters of importance, and with a willingness to give quick advice, in friendship and counsel—all this is of infinite service to the one in charge of the case.

"It would seem, therefore, that it is coming to be the function of the hospital to be the clearing house for obscure cases and cases of dire emergency; and chiefly in the care of the mass of people who are neither very rich nor extremely poor, for the two latter classes are already provided for."—W. E. F.

